# Sensor space group analysis for fNIRS data

**DOI:** 10.1016/j.jneumeth.2016.03.003

**Published:** 2016-05-01

**Authors:** S. Tak, M. Uga, G. Flandin, I. Dan, W.D. Penny

**Affiliations:** a Wellcome Trust Centre for Neuroimaging, University College London, 12 Queen Square, London WC1N 3BG, UK; b Jichi Medical University, Center for Development of Advanced Medical Technology, 3311-1 Yakushiji, Shimotsuke, Tochigi 329-0498, Japan; c Chuo University, Applied Cognitive Neuroscience Laboratory, 1-13-27 Kasuga, Bunkyo, Tokyo 112-8551, Japan

**Keywords:** Sensor space group analysis, Functional near-infrared spectroscopy, Random-effects analysis, Canonical scalp surface, Random field theory

## Abstract

**Background:**

Functional near-infrared spectroscopy (fNIRS) is a method for monitoring hemoglobin responses using optical probes placed on the scalp. fNIRS spatial resolution is limited by the distance between channels defined as a pair of source and detector, and channel positions are often inconsistent across subjects. These challenges can lead to less accurate estimate of group level effects from channel-specific measurements.

**New method:**

This paper addresses this shortcoming by applying random-effects analysis using summary statistics to interpolated fNIRS topographic images. Specifically, we generate individual contrast images containing the experimental effects of interest in a canonical scalp surface. Random-effects analysis then allows for making inference about the regionally specific effects induced by (potentially) multiple experimental factors in a population.

**Results:**

We illustrate the approach using experimental data acquired during a colour-word matching Stroop task, and show that left frontopolar regions are significantly activated in a population during Stroop effects. This result agrees with previous neuroimaging findings.

**Compared with existing methods:**

The proposed methods (i) address potential misalignment of sensor locations between subjects using spatial interpolation; (ii) produce experimental effects of interest either on a 2D regular grid or on a 3D triangular mesh, both representations of a canonical scalp surface; and (iii) enables one to infer population effects from fNIRS data using a computationally efficient summary statistic approach (random-effects analysis). Significance of regional effects is assessed using random field theory.

**Conclusions:**

In this paper, we have shown how fNIRS data from multiple subjects can be analysed in sensor space using random-effects analysis.

## Highlights

• We apply random-effects analysis using summary statistics to fNIRS data.
• Individual contrast images are generated in a 2D or 3D canonical scalp surface.
• Random-effects analysis then enables inference about population effects.
• We show that left frontopolar area is activated in a population during Stroop effects.
• Results are consistent with previous neuroimaging findings.

## 1 Introduction

Functional near-infrared spectroscopy (fNIRS) is a neuroimaging technique for monitoring hemodynamic and oxygenation changes in the brain by measuring changes in optical absorption (Jobsis, 1977, Villringer et al., 1993; for reviews, see Ferrari and Quaresima, 2012). Near-infrared light is transmitted to the surface of the scalp by optical fibres, and propagates several centimeters through tissue. The loss of light levels is then measured by optical detectors, and used to calculate the changes in hemoglobin concentrations in the underlying brain regions (Delpy et al., 1988). Although fNIRS has higher temporal resolution than functional magnetic resonance imaging (fMRI), its spatial resolution is limited by the optical source-detector distance which is typically 2–3 cm.

The poor spatial resolution of fNIRS leads to difficulties in sensor space analyses of multisubject fNIRS studies. Channel-wise group analysis is used to assess whether regionally specific effects are common across or between groups of interest, assuming that the channel positions are consistent across subjects (Ciftci et al., 2007, Ciftci et al., 2008). However, this assumption is often violated, because variability in head shape and size makes placement of the optical source and detector less reproducible (Tsuzuki and Dan, 2014). To address the misalignment of channel locations between subjects, several studies applied spatial interpolation to the channel-specific estimates from the individual subject analyses, and then performed group analysis on the individual topographic images (Schroeter et al., 2004, Plichta et al., 2007, Ye et al., 2009, Fekete et al., 2011, Tak and Ye, 2014).

Specifically, Fekete et al. (2011) used the two-level mixed-effects general linear model (GLM) (Beckmann et al., 2003) for the group analysis of interpolated fNIRS topographic maps. Spatial interpolation produces spatially correlated voxels, and it is therefore required to adjust *p*-value for the multiplicity of test performed. However, spatial correlation due to interpolation was not accommodated, when making inferences about regional effects at the group level. This problem can be solved by using random field theory (RFT) which allows one to assign adjusted *p*-values to topological features of random field statistics (Worsley et al., 1996). Tak and Ye (2014) used the two-level mixed-effects general linear mixed model (GLMM) (Searle, 1979, Cnaan et al., 1997) to relate the effects of interest at the group level to the concatenated individual fNIRS data, and its statistical significance was assessed using RFT. The GLMM makes fewer assumptions in estimating the error variances than the summary statistic approach that has been widely used in the group analysis of neuroimaging data (Holmes and Friston, 1998, Mumford and Nichols, 2009). However it is more computationally demanding, and this is particularly acute for fNIRS time series as they are acquired at higher sample rate than fMRI. Moreover, the group analysis presented in Tak and Ye (2014) was tested using smaller sample size (3 subjects) than the size typically used in functional neuroimaging studies (Desmond and Glover, 2002), and therefore further validation of the methods is required.

In this paper, as a practical solution to these issues, we apply random-effects analysis using summary statistics to fNIRS topographic data, to make inferences about regionally specific effects induced by (potentially) multiple experimental factors. In multisubject studies, random-effects analysis treats subject effects as random variables, and therefore allows for inferences about the population from which the subjects were drawn (Penny and Holmes, 2006). Random-effects analysis using the summary statistics approach has been adopted for group analysis of various neuroimaging data, including fMRI, electroencephalography (EEG), and magnetoencephalography (MEG) (Penny and Holmes, 2006, Litvak et al., 2011). It is a straightforward, computationally simple and flexible approach that can accomodate a broad range of experimental designs. Most simply, the experimental effect of interest can be captured using a single (summary) contrast image per subject and its statistical significance assessed using a one-sample *t*-test design at the second level. The generality of this approach stems from the fact that the summary images themselves can capture main effects or interaction effects from factorial designs (Winer et al., 1991, Penny and Henson, 2006).

In this paper, we show how random-effects analysis is applied to sensor space fNIRS data, focusing on (i) the generation of spatially interpolated contrast images for individual subjects, (ii) the implementation of group analysis pipeline which enables one to use the established random-effects analysis with the combined estimates from a group of subjects. In particular, we describe spatial interpolation methods for generating fNIRS contrast images either on a two-dimensional (2D) regular grid or on a three-dimensional (3D) triangular mesh, both representations of canonical scalp surface (Perrin et al., 1989, Litvak et al., 2011), and discuss under what circumstances each of these approaches is appropriate. Their topological features are then assessed in a statistical framework, when making inference about regionally specific effects at the group level (Taylor and Worsley, 2007, Kilner and Friston, 2010, Flandin and Friston, 2015). The proposed methods for the sensor space group analysis are validated using fNIRS data acquired from 21 subjects recorded during a colour-word matching Stroop task in an event-related design (Stroop, 1935, MacLeod, 1991).

This paper is structured as follows. In Section 2, we first describe a generative model of sensor space fNIRS data, and review the random-effects analysis via summary statistics approach. We then illustrate basic procedures by applying the random-effects analysis to fNIRS topographic images, with a special focus on issues that relate to spatial interpolation on the canonical scalp surface. In Section 3, we provide an illustrative group analysis using fNIRS data acquired during a colour-word matching Stroop task (Stroop, 1935, MacLeod, 1991).

## 2 Methods

Multiple-subject fNIRS data are analysed in a two-level process. In the first-level analysis, the general linear models for each subject are independently fitted to individual data, and in the second-level analysis, the population effects are estimated using the summary-statistics approach. The following subsections describe the fNIRS measurement model in sensor space, and then introduces within- and between-subject models for the group analysis of fNIRS data.

### 2.1 fNIRS measurement model in sensor space

Optical attenuation in a highly scattering medium is described by the following modified Beer–Lambert law (Delpy et al., 1988)

(1) $$\Delta \phi_{i,j}(\lambda )=(\alpha_{H}(\lambda )\Delta H_{i,j}+\alpha_{Q}(\lambda )\Delta Q_{i,j})d(\lambda ,a)l,$$

where *i* = 1, …, *M* observations, *j* = 1, …, *J* channels, *λ* is a particular wavelength, Δ*ϕ* is optical density change, Δ*H* and Δ*Q* are changes in oxygenerated and deoxygenated hemoglobin (HbO and HbR, [mM]), *α*_*H*_ and *α*_*Q*_ are the molar absorption coefficients [mM^−1^ cm^−1^] for HbO and HbR (Matcher et al., 1995), *d* is a differential pathlength factor (DPF) (Duncan et al., 1996, Scholkmann and Wolf, 2013) which depends on *λ* and age of subject *a*, and *l* is distance between optical source and detector [cm]. This modified Beer–Lambert law shows that the optical density changes are linearly proportional to the changes in absorption coefficients, reflecting the hemoglobin concentration changes. Measurements of optical density changes at two wavelengths can then be used to calculate the changes in HbO and HbR in underlying brain regions:

(2) $$\begin{array}{c} \Delta H_{i,j}=\frac{1}{\mathrm{lc}}\left(\frac{\alpha_{Q}(\lambda_{2})}{d(\lambda_{1},a)}\Delta \phi_{i,j}(\lambda_{1})-\frac{\alpha_{Q}(\lambda_{1})}{d(\lambda_{2},a)}\Delta \phi_{i,j}(\lambda_{2})\right),\\ \Delta Q_{i,j}=-\frac{1}{\mathrm{lc}}\left(\frac{\alpha_{H}(\lambda_{2})}{d(\lambda_{1},a)}\Delta \phi_{i,j}(\lambda_{1})-\frac{\alpha_{H}(\lambda_{1})}{d(\lambda_{2},a)}\Delta \phi_{i,j}(\lambda_{2})\right), \end{array}$$

where *c* = *α*_*H*_(*λ*_1_)*α*_*Q*_(*λ*_2_) − *α*_*H*_(*λ*_2_)*α*_*Q*_(*λ*_1_).

### 2.2 Within-subject model

The GLM for each subject can be written as (Friston et al., 1995, Ye et al., 2009)

(3) Y=Xβ+ϵ,

where *Y* is [*M* × *J*] matrix containing hemoglobin responses (Δ*H* or Δ*Q*) from a particular subject, *M* is total number of scans, and *J* is total number of channels; *X* is [*M* × *L*] design matrix containing *L* regressors of interest (e.g., stimulus function convolved by the canonical hemodynamic response function) and confounds; *β* is [*L* × *J*] matrix of regression coefficients; and *ϵ* is [*M* × *J*] matrix of zero-mean normally distributed errors. The variance of the errors at a particular channel is given by *σ*^2^*V*, where *σ*^2^ is channel-specific and *V* is a global temporal autocorrelation matrix.

An estimator of channel-specific parameter *β* can be obtained by multiplying the observations *Y* and their model by a filter matrix *S* then using the least squares:

(4) $$\hat{\beta }={\left(X^{*T}X^{*}\right)}^{-1}X^{*T}Y^{*},$$

where X^*^ = *SX*, Y^*^ = *SY*, and a filter matrix is set to *S* = *V*^−(1/2)^ for whitening the data before fitting the GLM. The intrinsic temporal correlation *V* can be estimated, based on a first order autoregressive model (AR(1)) (Purdon and Weisskoff, 1998), and its model parameters are estimated using a restricted maximum likelihood (ReML) method (Friston et al., 2002).

The effects of interest are then estimated as $c^{T}\hat{\beta }$, where *c* is [*L* × 1] contrast vector which forms a linear combination of parameter estimates.

### 2.3 Group analysis via summary-statistics

Random-effects analysis takes into account within-subject and between-subject variability, and allows for making inferences about population effects (Penny and Holmes, 2006). For a given location, this random effects analysis is implemented using the summary-statistics approach:

(5) $$\begin{array}{c} {\bar{w}}_{n}=w_{n}+e_{n},\\ w_{n}=w_{\mathrm{pop}}+z_{n}, \end{array}$$

where *n* = 1, …, *N* subjects; $w_{n}=c^{T}\beta_{n}$ is the true mean effect for subject *n* at a particular location; ${\bar{w}}_{n}=c^{T}{\hat{\beta }}_{n}$ is the sample mean effect; $w_{\mathrm{pop}}=X_{n,G}\beta_{G}$ is the true effect for the population, where *X*_*n*,*G*_ is the [1 × *P*] row vector in the group-level design matrix, *β*_*G*_ is the [*P* × 1] vector of group-level parameters; the within-subject Gaussian error *e*_*n*_ has zero mean and variance $\sigma_{w}^{2}$; and the between-subject Gaussian error *z*_*n*_ has zero mean and variance $\sigma_{b}^{2}$. Collapsing the two levels into one gives

(6) $${\bar{w}}_{n}=w_{\mathrm{pop}}+e_{n}^{*},$$

where the mixed-effects error, $e_{n}^{*}=e_{n}+z_{n}$, has zero mean and a variance given by

(7) $$\mathrm{Var}[e_{n}^{*}]=\mathrm{Var}[e_{n}]+\mathrm{Var}[z_{n}]=\sigma_{w}^{2}+\sigma_{b}^{2}.$$

Thus we see that, on average, the error variance contains a contribution from both the within and between subject errors. Considering Eq. (6) for all subjects gives

(8) $$\bar{W}=X_{G}\beta_{G}+E^{*},$$

where

(9) $$\bar{W}=\left[\begin{array}{c} c^{T}{\hat{\beta }}_{1}\\ c^{T}{\hat{\beta }}_{2}\\ \vdots \\ c^{T}{\hat{\beta }}_{N} \end{array}\right],\;X_{G}=\left[\begin{array}{c} X_{1,G}\\ X_{2,G}\\ \vdots \\ X_{N,G} \end{array}\right],\;E^{*}=\left[\begin{array}{c} e_{1}^{*}\\ e_{2}^{*}\\ \vdots \\ e_{N}^{*} \end{array}\right].$$

The least squares estimate of *β*_*G*_ is then given by

(10) $${\hat{\beta }}_{G}={\left(X_{G}^{T}X_{G}\right)}^{-1}X_{G}^{T}\bar{W}=X_{G}^{-}\bar{W},$$

and its variance is estimated as

(11) $$\begin{array}{c} \hat{\mathrm{Var}}[{\hat{\beta }}_{G}]=X_{G}^{-}\hat{\mathrm{Var}}[E^{*}]X_{G}^{-T},\\ \hat{\mathrm{Var}}[E^{*}]={\left(\bar{W}-X_{G}\hat{\beta_{G}}\right)}^{T}(\bar{W}-X_{G}\hat{\beta_{G}})/(N-P). \end{array}$$

### 2.4 Individual topographic images

In Eq. (4), we have estimated the GLM regression coefficient, $\hat{\beta }$, and the effects of interest, $c^{T}\hat{\beta }$ for each subject from fNIRS channel measurements. However, the spatial resolution of the channel-specific estimates is limited to the optical source-detector distance due to the high level of light scattering. Additionally, optical probe locations are not consistent across subjects due to variability in head shape and size. It is therefore necessary to estimate (i.e. interpolate) the effects of interest for each subject at intervening voxels. Spatial interpolation of the channel-specific estimates on the canonical scalp surface generates individual topographic images containing the voxel-specific effects of interest, $c^{T}\hat{\beta }(r)$ where *r* is a voxel location. The implied smoothing in this interpolation blurs effects that are focal in space, and ensures overlap among a group of subjects.

Specifically, individual topographic images can be computed in two stages. Channel positions are first normalised to the Montreal Neurological Institute (MNI) coordinate system using a virtual registration method (Okamoto et al., 2004, Singh et al., 2005, Tsuzuki et al., 2007), and projected either onto a 2D regular grid or onto a 3D triangular mesh, both representations of canonical scalp surface. Surface interpolations for scattered data on 2D and 3D canonical scalp surfaces are then applied to channel-specific estimates of GLM parameters, to generate individual topographic images.

In the topographic mapping on a 2D regular grid (Kiebel and Friston, 2004), we perform the linear interpolation on a planar and circular surface that accords with the international 10–20 system and is commonly used in EEG/MEG, and fNIRS data displays (Jasper, 1958, Jurcak et al., 2007, Litvak et al., 2011). The 2D topographic image is then smoothed by multidimensional convolution with a Gaussian kernel, to accommodate spatial variability over subjects and ensure the images conform to the assumptions of the topological inference approach (RFT applies to continuous statistical processes and we must ensure the smoothness of the underlying statistical field is large in relation to the voxel size (Friston et al., 2007)). The image is saved in standard NIfTI-1 data format prior to group level analysis.

In the topographic mapping on a 3D triangular mesh (Mattout et al., 2007), spherical splines are applied to construct spatial maps of the effects of interest for each subject (Perrin et al., 1989, Oostenveld et al., 2010). Specifically, the scalp channel positions are projected on the sphere and represented using spherical coordinates (Estrin and Uzgalis, 1969). The effects of interest at any position *r*_*i*_ on the canonical scalp surface is then given by

(12) $$\begin{array}{c} c^{T}\hat{\beta }(r_{i})=k_{0}+\sum_{j=1}^{J}k_{j}g(\cos(r_{i},r_{j,c})),\\ g_{m}(x)=\frac{1}{4\pi }\sum_{n=1}^{\infty }\frac{n^{m}{\left(n+1\right)}^{m}}{2n+1}P_{n}(x), \end{array}$$

where *r*_*j*,*c*_ is the *j*th channel position, *j* = 1, …, *J*; cos(*r*_*i*_, *r*_*j*,*c*_) is the cosine of the angle between interpolation position *r*_*i*_ and channel position *r*_*j*,*c*_; *k*_0_ and *k*_*j*_ are coefficients fit to the data; *P*_*n*_ is the *n*th degree Legendre polynomial; *m* is the spline order which controls the level of smoothness of interpolation kernel, and a value of *m* = 3 or 4 is typically chosen (Ferree, 2006). The *J* + 1 coefficients *k*_*j*_ are calculated by imposing two conditions (i) sum of coefficients *k*_*j*_ is equal to zero, and (ii) the interpolation function should reproduce the data when evaluated at the channels:

(13) $$\begin{array}{c} \sum_{j=1}^{J}k_{j}=0,\\ c^{T}\hat{\beta }(r_{j,c})=k_{0}+\sum_{k=1}^{J}k_{j}g(\cos(r_{j,c},r_{k,c})). \end{array}$$

The 3D topographic image on the canonical scalp surface is then saved in standard surface-based data format GIfTI.

### 2.5 Colour-word matching Stroop task data

We now apply the summary statistics approach to random effects analysis to sensor space fNIRS data from 21 subjects. Twenty-six healthy subjects participated in this study (range 23–63 years, mean = 41.3, SD = 14.5, 14 males, 12 females). Among these participants, 3 subjects were excluded from the study before the data were collected due to failure to accomplish experimental tasks, and 2 subjects were excluded from the group analysis due to excessive artifacts in data. All subjects gave informed written consent prior to testing under the approval of the Jichi Medical University ethics committee.

**Fig. 1:**
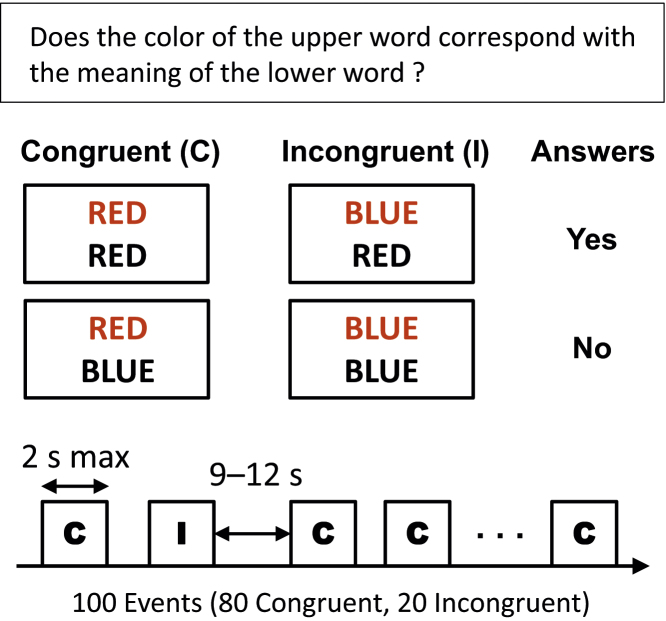
Example of congruent and incongruent conditions of the colour-word matching Stroop task. Subjects were instructed to determine whether the colour of the top row letters corresponded to the colour name written on the bottom row. In the congruent condition, the colour word at the top row was presented in the congruent colour, whereas for the incongruent condition, the colour word was presented in a different colour. Stimuli were presented randomly in 80 congruent and 20 incongruent combinations of four colour words “red”, “blue”, “yellow”, and “green”. Each stimulus was presented until a response was given, with a maximum allowed response time of 2 s. Interstimulus interval was randomly selected in a range of 9–12 s. One of the objectives of this study was to understand brain activity associated with the suppression of automatic responses (Stroop, 1935, MacLeod, 1991). (For interpretation of the references to colour in this figure legend, the reader is referred to the web version of this article.)

fNIRS data were recorded during a colour-word matching Stroop task in an event-related design, under congruent and incongruent experimental conditions (Stroop, 1935, MacLeod, 1991). Subjects wearing a cap that holds the optical probes were seated in front of a computer screen on which two rows of letters were displayed. The subjects were instructed to determine whether the colour of the top row letters corresponded to the colour name written on the bottom row. In the congruent condition, the colour word at the top row was presented in the congruent colour, whereas for the incongruent condition, the colour word was presented in a different colour, as shown in Fig. 1. Stimuli were presented randomly in 80 congruent and 20 incongruent combinations of four colour words “red”, “blue”, “yellow”, and “green”. Each stimulus was presented until a response was given, with a maximum allowed response time of 2 s. Interstimulus interval was randomly selected in a range of 9–12 s. One of the objectives of this study was to understand brain activity associated with the suppression of automatic responses. Previous studies showed activations in middle frontal gyrus (MFG), inferior frontal gyrus (IFG), and anterior cingulate cortex while performing the Stroop task (MacLeod and MacDonald, 2000, Leung et al., 2000).

**Fig. 2:**
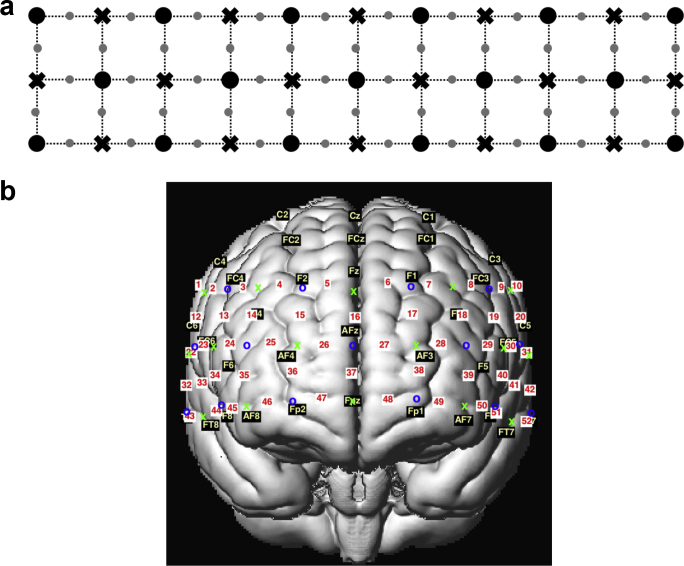
(a) Geometry of optical probes covering the prefrontal area, and (b) results of spatial preprocessing. ‘o’ and ‘x’ indicate optical source and detector. ‘number’ on white background indicates a functional near-infrared spectroscopy (fNIRS) channel position defined as a midpoint between a source and detector. The channel positions are transformed into the Montreal Neurological Institute (MNI) space, and then projected onto the surface of a volume rendered brain (Okamoto et al., 2004, Singh et al., 2005). ‘letter + number’ on black background indicates an electroencephalography (EEG) electrode position in the 10–10 system (Chatrian et al., 1985, Jurcak et al., 2007), an extension to the international 10–20 system (Jasper, 1958).

Optical density changes during the above Stroop task were acquired using a multichannel fNIRS optical topography system (ETG-4000, Hitachi Medical Corporation, Kashiwa, Japan). The fNIRS system had 52 channels, consisting of 17 optical sources with wavelengths of 695 nm and 830 nm, and 16 optical detectors. The sampling frequency was 10 Hz. The distance between the optical source and detector was 3 cm. The geometry of the optical probes covering the prefrontal area is shown in Fig. 2. EEG electrode positions in the 10–10 system (Chatrian et al., 1985, Jurcak et al., 2007) are additionally displayed in Fig. 2(b), to provide the standard cranial landmarks.

The first-level (individual subject) analysis was performed with the SPM for fNIRS toolbox (https://www.nitrc.org/projects/spm_fnirs/). Specifically, the hemoglobin concentration changes for each channel were calculated from fNIRS data using the modified Beer–Lambert law (Delpy et al., 1988). Time series of hemoglobin changes were preproprocessed with the following steps: (i) motion artifact was reduced using a method based on moving standard deviation and spline interpolation (Scholkmann et al., 2010); (ii) physiological noise, including respiration and cardiac pulsation, was removed using a band-stop filter with stopband frequencies of 0.12–0.35 Hz and 0.7–2.0 Hz, respectively. In both cases, we used a fifth-order infinite impulse response (IIR) Butterworth filter (Oostenveld et al., 2010); and (iii) very low-frequency confounds were removed using a high-pass filter based on a discrete cosine transform set with a cutoff frequency of 1/64 Hz. The data were downsampled to 1 Hz and whitened using the AR (1) model (Purdon and Weisskoff, 1998, Friston et al., 2002). In this study, fNIRS data was recorded using optical source and detector separated by 3 cm, and therefore hemoglobin response analysed from each channel measurement contained signals arising from both cerebral and extracerebral compartments (i.e. cerebral blood flow and scalp blood flow) (Hirasawa et al., 2015, Selb et al., 2014). This would result in reduced sensitivity of fNIRS to changes in the cortex versus changes in the extracerebral tissue. However, when a short source-detector separation channel (sensitive to superficial layers only) is available, extracerebral effects can be reduced and separated from the fNIRS signal using state-space modelling with Kalman filter (Gagnon et al., 2011) and independent component analysis (Funane et al., 2014).

In the spatial preprocessing step, channel positions in subject space were transformed into corresponding positions in MNI space using the NFRI functions (Okamoto et al., 2004, Singh et al., 2005). The GLM for each subject was then fitted to the channel-specific responses. The first-level design matrix was constructed to include two regressors modelling two conditions (congruent and incongruent events) convolved with a basis set consisting of the canonical hemodynamic response function and its temporal derivative, which allowed to take into account of the variability in the delay of the peak of the response. In our approach, the canonical hemodynamic function was characterised by a double gamma function (onset delay, 0 s; peak delay, 6 s; undershoot delay, 16 s) (Boynton et al., 1996, Friston et al., 1998). However, it is also possible to utilise an adaptive hemodynamic response function in the current framework, to adjust parameters for temporally different behaviours of HbO and HbR (Uga et al., 2014). Overall, the first-level analysis allowed us to estimate the effects of interest for each subject, defined with the relevant contrasts of the GLM parameter estimates, from the channel-specific fNIRS response.

**Fig. 3:**
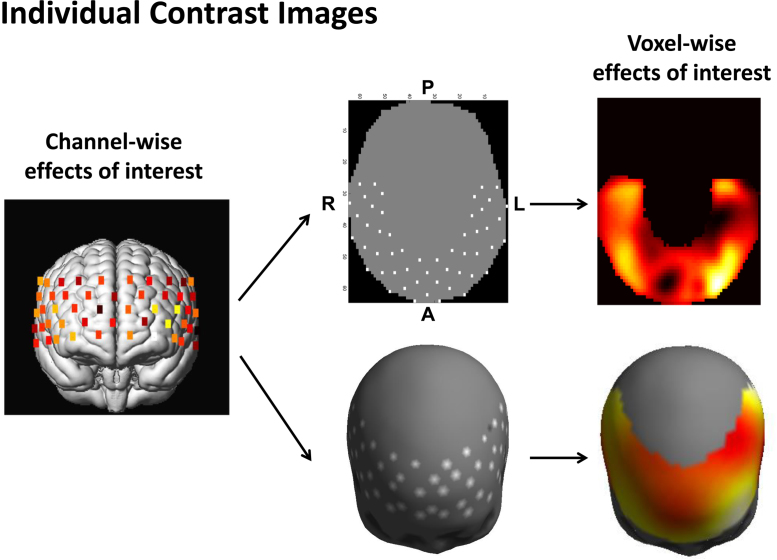
Procedure for computing the individual contrast images per subject from channel-wise estimates of general linear model (GLM) parameters. The GLM regression coefficients and the effects of interest (contrast) for each subject are estimated from fNIRS channel measurements, and then projected onto either a two-dimensional (2D) regular grid or a three-dimensional (3D) triangular mesh, both representations of canonical scalp surface. Spatial interpolation of the channel-specific estimates on the canonical scalp surface generates individual contrast images (Perrin et al., 1989, Oostenveld et al., 2010, Litvak et al., 2011). The implied smoothing in this interpolation blurs effects that are focal in space, and ensures overlap among a group of subjects.

Individual topographic images for each of the contrasts were made by interpolating the channel-wise contrast on the 2D regular grid (64 pixels in each spatial direction), and 3D triangular mesh (2562 vertices), both representations of canonical scalp surface. Specifically, 2D contrast images were produced by using linear interpolation between channels. The images were smoothed using a Gaussian kernel with full width at half maximum (FWHM) of 14 mm, to make the error field a reasonable lattice approximation of a random field with a multivariate Gaussian distribution. In 3D topographic mapping, spherical splines with the order of 4 were applied to the channel-wise contrasts. The procedures for computing 2D and 3D contrast images per subject are summarised in Fig. 3.

**Fig. 4:**
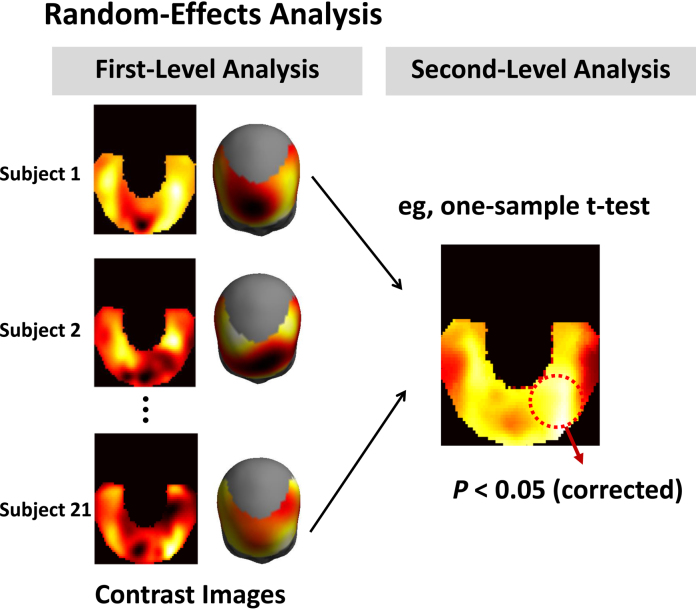
Schematic diagram describing the random-effects analysis using summary-statistics for functional near-infrared spectroscopy (fNIRS). The experimental effect of interest is captured using a single contrast image per subject, and its statistical significance is assessed using a one-sample *t*-test design at the second level (Penny and Holmes, 2006). The generality of this approach stems from the fact that the summary images themselves can capture main effects or interaction effects from factorial designs (Winer et al., 1991, Penny and Henson, 2006).

The contrast images of all subjects from the first-level were then analysed as a random-effects analysis via summary-statistics implemented in SPM12 (Penny and Holmes (2006), http://www.fil.ion.ucl.ac.uk/spm/software/spm12/). The random-effects analysis allowed fNIRS topographic images to be used for making inference about the population from which subjects were drawn. A schematic diagram describing the random-effects analysis using summary-statistics for fNIRS is shown in Fig. 4.

## 3 Results

**Fig. 5:**
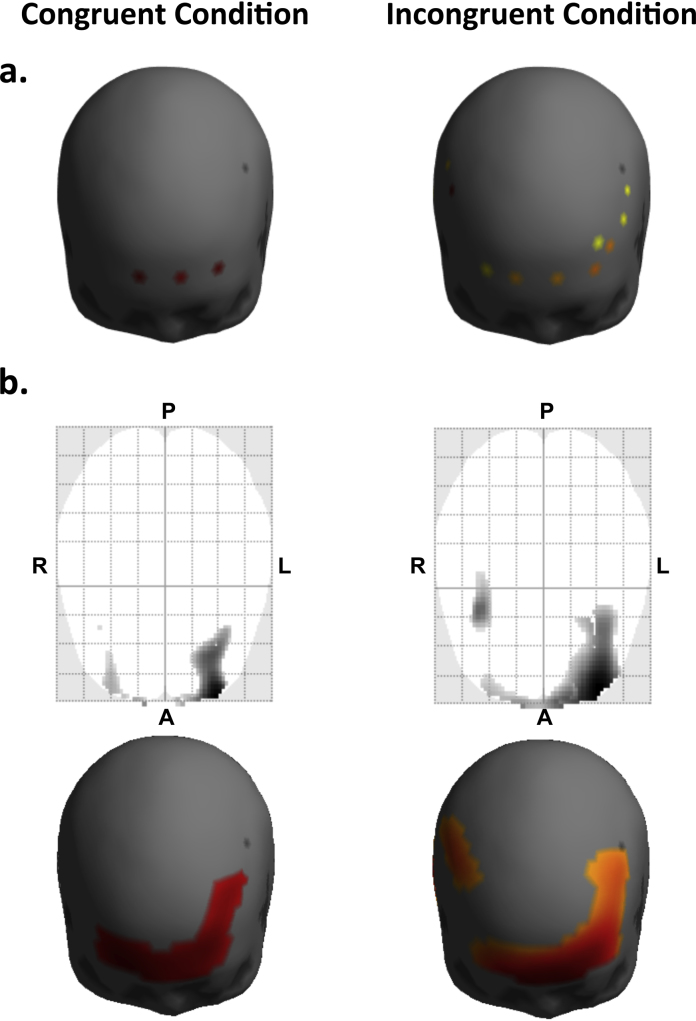
Effects of congruent and incongruent conditions in a population. The statistical significance of each effect in the population was assessed using one-sample *t*-tests at the second level. The resulting statistical parametric maps were thresholded at *p* < 0.02. The *p*-value was corrected for multiple comparisons over (a) all sensors using the Bonferroni correction, and (b) all voxels/vertices on the canonical scalp surface (search region) using random field theory (RFT) (Taylor and Worsley, 2007, Friston et al., 2007, Friston et al., 1994, Kilner and Friston, 2010, Flandin and Friston, 2015). The cluster-forming height thresholds according to RFT ($T_{c}^{2D}=3.74$, $T_{c}^{3D}=3.66$) were lower than the Bonferroni threshold (*T* = 3.96). The RFT was therefore more sensitive than the Bonferroni correction, which allowed us to observe more activation during the Stroop task. We observed a significant cluster containing bilateral frontopolar area and left inferior/middle frontal areas during the incongruent condition ($p_{\mathrm{FWE}}^{2D}<0.0002$, $p_{\mathrm{FWE}}^{3D}<0.0003$). Maximum *t*-value in each cluster was located at the left middle frontal area, and the channel nearest to the peak was 48 ($p_{\mathrm{FWE}}^{2D}<0.002$, $p_{\mathrm{FWE}}^{3D}<0.001$). A similar pattern of activation was observed during the congruent condition, albeit with fewer voxels significantly activated. *p*_FWE_ denotes the family-wise error rate corrected over the search region.

Effects of the colour-word matching Stroop task were identified in a population using random-effects analysis, as shown in Fig. 5. Two contrast images per subject, containing the effects of congruent and incongruent conditions, were computed from the HbO response by applying spatial interpolation to the channel-wise contrast values. The statistical significance of each effect in the population was then assessed using one-sample *t*-tests at the second level. The degrees of freedom for these tests are *df* = 20.

The statistical parametric maps (SPMs) were thresholded using RFT (Taylor and Worsley, 2007, Friston et al., 2007). These results were compared to results obtained from conventional procedures by analysing data from each channel separately and then applying a Bonferroni correction (Ciftci et al., 2008, Schroeter et al., 2002, Fekete et al., 2011). Fig. 5 shows *T*-statistic maps thresholded at *p* < 0.02, correcting for multiple comparisons over (a) all channels using the Bonferroni correction, and (b) all voxels/vertices on the canonical scalp surface (search region) using RFT. The search region contained 1609 voxels for 2D SPMs and 291 vertices for 3D SPMs. The cluster-forming height thresholds according to RFT ($T_{c}^{2D}=3.74$, $T_{c}^{3D}=3.66$) were lower than the Bonferroni threshold (*T* = 3.96). Therefore, the RFT was more sensitive than the Bonferroni correction, which allowed us to observe more activation during the Stroop task.

In the context of the RFT, topological features of the excursion set were assessed controlling the family-wise false positive rate of peaks (a local maximum) or clusters (a connected component of the excursion set) (Friston et al., 1994, Flandin and Friston, 2015). We observed a significant cluster containing bilateral frontopolar area and left inferior/middle frontal areas during the incongruent condition ($p_{\mathrm{FWE}}^{2D}<0.0002$, $p_{\mathrm{FWE}}^{3D}<0.0003$), as shown in the right column of Fig 5b. Maximum *t*-value in each cluster was located at the left middle frontal area, and the channel nearest to the peak was 48 ($p_{\mathrm{FWE}}^{2D}<0.002$, $p_{\mathrm{FWE}}^{3D}<0.001$), where *p*_FWE_ denotes the family-wise error rate corrected over the search region. A similar pattern of activation was observed during the congruent condition, albeit with fewer voxels significantly activated, as shown in the left column of Fig. 5b.

Details of significance thresholds and peak/cluster-level inferences are summarised in Tables 1 and 2. Importantly, the number of resolution elements (RESELS) in the search region is very similar for 2D versus 3D SPMs (Taylor and Worsley, 2007, Friston et al., 2007).

**Table 1:** Statistical results of effects of congruent condition. Random field theory computes the cluster-forming height threshold *T*_*c*_ that gives a corrected *p*-value (for peaks) of *p* < 0.02. The size of each cluster is then the number of contiguous voxels/vertices above *T*_*c*_. We report the number of resolution elements (RESELS) in the search region, the number of voxels *k*_*E*_ in each cluster, the maximum *t*-value in each cluster, *T*, and the family-wise error rate of either cluster-level or peak-level effects, *p*_FWE−corr_.

			Cluster-level		Peak-level	
	RESELS	*T*_*c*_	*p*_FWE−corr_	*k*_E_	*p*_FWE−corr_	*T*
2D	2.5	3.74	0.005	133	0.01	4.10
3D	2.7	3.66	0.003	89	0.01	4.00

**Table 2:** Statistical results of effects of incongruent condition. Random field theory computes the cluster-forming height threshold *T*_*c*_ that gives a corrected *p*-value (for peaks) of *p* < 0.02. We report the number of resolution elements (RESELS) in the search region, the number of voxels *k*_*E*_ in each cluster, the maximum *t*-value in each cluster, *T*, and the family-wise error rate of either cluster-level or peak-level effects, *p*_FWE−corr_.

			Cluster-level		Peak-level	
	RESELS	*T*_*c*_	*p*_FWE−corr_	*k*_E_	*p*_FWE−corr_	*T*
2D	3.3	3.85	0.0002	314	0.002	4.91
3D	3.5	3.78	0.0003	140	0.001	5.19

Table 3

**Table 3:** Statistical results of effects of difference between incongruent and congruent conditions. Random field theory computes the cluster-forming height threshold *T*_*c*_ that gives a corrected *p*-value (for peaks) of *p* < 0.05. We report the number of resolution elements (RESELS) in the search region, the number of voxels *k*_*E*_ in each cluster, the maximum *t*-value in each cluster, *T*, and the family-wise error rate of either cluster-level or peak-level effects, *p*_FWE−corr_.

			Cluster-level		Peak-level	
	RESELS	*T*_*c*_	*p*_FWE−corr_	*k*_E_	*p*_FWE−corr_	*T*
2D	5.9	3.61	0.042	7	0.038	3.75
3D	6.4	3.55	0.011	32	0.005	4.80

**Fig. 6:**
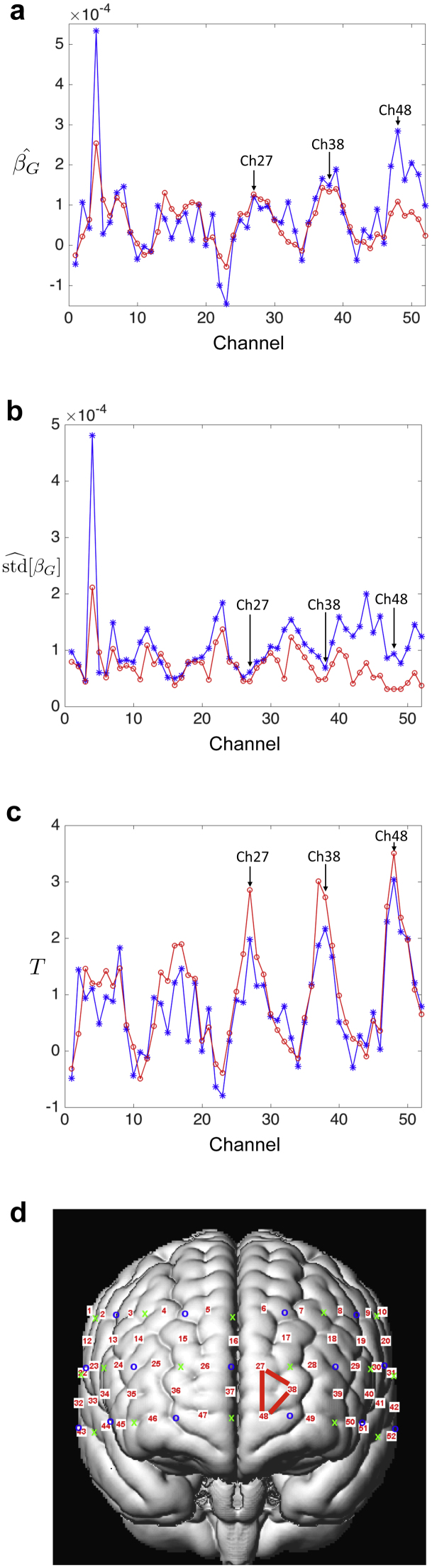
Group-level parameters at individual sensors. (a) The least-square estimate of *β*_*G*_ in Eq. (10), (b) the standard deviation of $\hat{\beta_{G}}$ in Eq. (11), (c) the corresponding *T*-values, and (d) the channel positions on the surface of a volume rendered brain. The blue plots indicate the group level parameters obtained from conventional procedures by analysing data from each channel separately. The red plots indicate the group level parameters obtained from the proposed method by analysing data from spatially interpolated contrast images. The implied spatial smoothing in the 2D and 3D SPMs significantly reduced standard deviation of group-level parameters over subjects ($\hat{\text{std}}[{\hat{\beta }}_{G}]$), which led to increases in *T*-values at individual sensors and nearby voxels in 2D and 3D SPMs. (For interpretation of the references to colour in this figure legend, the reader is referred to the web version of this article.)

A one-sample *t*-test was additionally performed (*df* = 20), to make an inference about the difference between incongruent and congruent effects in the population. The resulting SPM was thresholded at *p* < 0.05, correcting for multiple comparisons over all channels using the Bonferroni correction, and over all voxels/vertices on the canonical scalp surface (search region) using RFT. The Bonferroni threshold (*T* = 3.57) was intermediate between the RFT thresholds for 2D and 3D SPMs ($T_{c}^{2D}=3.61$, $T_{c}^{3D}=3.55$). However, the implied spatial smoothing in the 2D and 3D SPMs significantly reduced standard deviation of group-level parameters over subjects ($\hat{\text{std}}[{\hat{\beta }}_{G}]$), which led to increases in *T*-values at individual sensors and nearby voxels in 2D and 3D SPMs (see Fig. 6). Therefore, although the largest detected *T*-value at individual sensors did not exceed the Bonferroni threshold, we observed significantly activated voxels surrounded by sensors 27, 39, and 48 after thresholding using RFT. Overall, the RFT approach is attractive in that it automatically adjusts for the statistical dependencies in the data. This is reflected, for example, in the RESEL count with more RESELS for the difference test (e.g., 6.4 for 3D reflecting rougher images) than for the individual conditions (e.g., 3.5 for 3D incongruent condition).

**Fig. 7:**
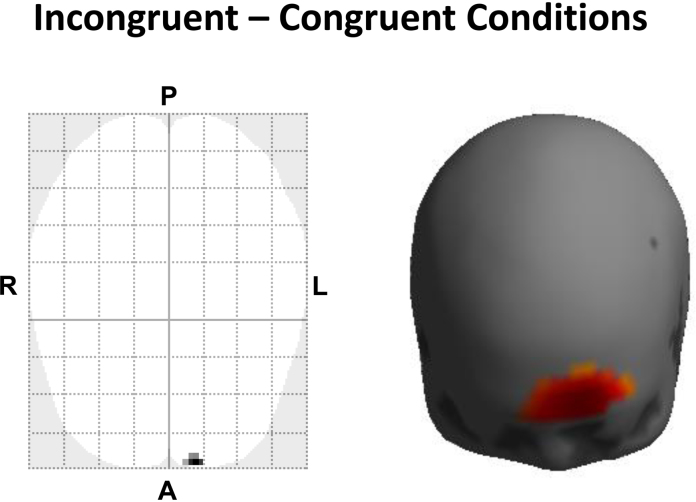
Effects of Stroop interference (incongruent–congruent conditions) in a population. A one-sample *t*-test was performed, to make inference about the difference between incongruent and congruent effects in the population. The resulting statistical parametric map was thresholded at *p* < 0.05, correcting for multiple comparisons over all voxels/vertices on the canonical scalp surface (search region). A significant cluster was observed in the left frontopolar area ($p_{\mathrm{FWE}}^{2D}<0.042$, $p_{\mathrm{FWE}}^{3D}<0.011$). Maximum *t*-value in each cluster was located at the left frontopolar area, and the channel nearest to the peak was 48 ($p_{\mathrm{FWE}}^{2D}<0.038$, $p_{\mathrm{FWE}}^{3D}<0.005$). These results indicate that activation in the left frontopolar area was significantly stronger during incongruent than congruent conditions, and agrees with previous neuroimaging findings (Schroeter et al., 2002, Ehlis et al., 2005, Byun et al., 2014, MacLeod and MacDonald, 2000, Leung et al., 2000).

A cluster of significant effects was observed in the left frontopolar area ($p_{\mathrm{FWE}}^{2D}<0.042$, $p_{\mathrm{FWE}}^{3D}<0.011$), as shown in Fig. 7. Maximum *t*-value in each cluster was located at the left frontopolar area, and the channel nearest to the peak was 48 ($p_{\mathrm{FWE}}^{2D}<0.038$, $p_{\mathrm{FWE}}^{3D}<0.005$). These results indicate that activation in the left frontopolar area was significantly stronger during incongruent than congruent conditions. Our results correspond to the findings of previous studies which have shown that left prefrontal regions, including frontopolar and inferior frontal cortices, were the most strongly activated area occurred for the incongruent−congruent contrast, while bilateral activation in the frontopolar and inferior/middle frontal regions were observed during the activity in each of incongruent and congruent conditions individually (Schroeter et al., 2002, Ehlis et al., 2005, Byun et al., 2014, MacLeod and MacDonald, 2000, Leung et al., 2000).

## 4 Discussion

In this paper, we have shown how fNIRS data from multiple subjects can be analysed in sensor space using random-effects analysis (Penny and Holmes, 2006) widely used in various neuroimaging modalities including fMRI, PET, and MEG. The experimental effects of interest are captured using one or more contrast images per subject, and their statistical significance over the population assessed using the summary statistics approach to random-effects analysis. We illustrated the methods using fNIRS data recorded during a colour-word matching Stroop task (*n* = 21 subjects). Specifically, by applying random-effects analysis to fNIRS data, we showed that left frontopolar regions were significantly activated in the population for incongruent−congruent contrast in a colour-word matching Stroop task. This result agrees with previous neuroimaging studies using fMRI, PET, and fNIRS that have previously detected regionally specific effects in the prefrontal area related to Stroop effects (Schroeter et al., 2002, Ehlis et al., 2005, Byun et al., 2014, MacLeod and MacDonald, 2000, Leung et al., 2000).

We have generated sensor space contrast images on the 2D and 3D canonical scalp surfaces (Jasper, 1958, Litvak et al., 2011). Linear interpolation with Gaussian spatial smoothing and spherical splines were used in 2D and 3D topographic mapping respectively, to accommodate potential misalignment of channel locations and spatial variability over subjects (Perrin et al., 1989, Oostenveld et al., 2010, Litvak et al., 2011). The 3D approach may be perceived as a more natural space in which to view the data as it provides a better reflection of the underlying physical reality (i.e. sensors are located on a 2D manifold in a 3D space) (Cutini et al., 2011). The viewpoint can be changed by rotating the 3D canonical scalp surface on the screen. This allows the user to select a specific view showing where experimental effects mainly appear (note that statistical results are entirely independent of the viewpoint). The 2D approach displays experimental effects on a planar and circular surface that accords with the international 10–20 system. This has the benefit of being more familiar to users of, for example, EEG (Jasper, 1958), and does not require the user to additionally specify a point of view. Additionally, 2D contrast images are much quicker to generate than 3D contrast images. For example, on a desktop PC running Windows 8.1 (64 bit) with an Intel Xeon 3.0 GHz, a single 2D image takes 1–2 s, whereas a 3D image takes approximately 1 min.

We have applied the random-effects analysis using summary statistics to fNIRS topographic images, to make inference about the population effects (Holmes and Friston, 1998, Penny and Holmes, 2006). Statistical significance of regionally specific effects was then assessed using RFT (Taylor and Worsley, 2007, Kilner and Friston, 2010, Flandin and Friston, 2015). Compared with the full mixed effects model, the summary statistic approach is computationally much simpler to implement, and gives identical results unless subjects have widely varying within-subject error variance or number of trials, whereas the full mixed effects model does not require assumptions of equal within-subject error variances (Penny and Holmes, 2006, Mumford and Nichols, 2009). These are the reasons why we applied classical summary statistic approach to fNIRS topographic images.

A previous fNIRS study introduced several group analysis methods for channel-specific fNIRS responses, and compared the results from summary statistic approaches with those obtained from Bayesian analysis of the concatenated individual fNIRS data (Ciftci et al., 2007, Ciftci et al., 2008). They showed that summary statistic approaches, including mixed-effects and random-effects analysis, gave similar results to the Bayesian approach (see Fig. 2 in Ciftci et al. (2008)) and within-subject error variance was much lower than between-subject error variance for fNIRS signals, which supports the validity of our approach.

Several fNIRS studies used the mixed-effects model for the group analysis of fNIRS topographic maps (Fekete et al., 2011, Tak and Ye, 2014). Specifically, Fekete et al. (2011) made inference about population effects from visual task data using a two-level mixed-effects model (Beckmann et al., 2003). However, RFT was not employed to control the false positive rate of topological features when making inference on spatially interpolated data. Tak and Ye (2014) applied a full mixed-effects model (Searle, 1979, Cnaan et al., 1997) to the concatenated individual fNIRS data. While the full mixed-effects model makes fewer assumptions in estimating the error variances than the summary statistic approach, equivalence between two-level and single-level mixed effects models would not hold for the interpolated parameter estimates. It is therefore more computationally demanding, and the analysis needs to be repeated for all subjects, if a new subject is included in a group of subjects.

Finally, we have focused on producing sensor space contrast images on a canonical scalp surface. While sensor space approaches enable us to infer regionally specific effects on the canonical scalp surface, several studies have also performed source reconstruction for generating depth-resolved images of the hemoglobin concentration changes, which is referred to as diffuse optical tomography (DOT) (Gibson et al., 2005, Abdelnour and Huppert, 2011, Hassanpour et al., 2014, Aasted et al., 2015). Specifically, Abdelnour and Huppert (2011) described the random-effects model for concatenated optical measurements, and source parameters at the group level were then estimated using the restricted maximum likelihood (ReML) method (Mattout et al., 2006). Hassanpour et al. (2014) applied SPM methods in the framework of general linear model (Friston et al., 2007) to high density DOT data, to make inference about regionally specific effects on the cortical regions with relatively high spatial resolution.

Although computation of contrast images in source space is beyond the scope of this work, it is worth noting that dynamic causal modelling (DCM) for fNIRS is a form of source space analysis (Friston et al., 2003, Tak et al., 2015). DCM-fNIRS is based on forward models relating optical measurements to hemodynamic activities and underlying neuronal interactions at specified point source locations. Inversion of these models, using an established Bayesian framework, then enables inference about regional activity and directed connectivity changes among source locations at the neuronal level (Friston et al., 2007b, Penny et al., 2010). For example, in the colour-word matching Stroop task, one might use DCM-fNIRS to investigate how a fronto-cingulate network including dorsolateral prefrontal cortex and rostral part of anterior cingulate cortex is modulated by the Stroop interference effect (Schlösser et al., 2008).

## Appendix A Software note

The first-level fNIRS analysis, including preprocessing, artefact removal, general linear modelling, and spatial interpolation routines were all implemented using the SPM for fNIRS toolbox. This is available from https://www.nitrc.org/projects/spm_fnirs/. Sensor-space contrast images can then be analysed using SPM12 in the usual way (SPM12-Manual, 2014).
